# Corrigendum

**DOI:** 10.3892/ol.2013.1141

**Published:** 2013-01-18

**Analysis of complete response by MRI following neoadjuvant chemotherapy predicts pathological tumor responses differently for molecular subtypes of breast cancer**

YUJI HAYASHI, HIROYUKI TAKEI, SATOSHI NOZU, YOSHIHIRO TOCHIGI, AKIHIRO ICHIKAWA, NAOKI KOBAYASHI, MASAFUMI KUROSUMI, KENICHI INOUE, TAKASHI YOSHIDA, SHIGENORI E. NAGAI, HANAKO OBA, TOSHIO TABEI, JUN HORIGUCHI and IZUMI TAKEYOSHI

Oncology Letters 5: 83–89, 2013; DOI: 10.3892/ol.2012.1004

After the publication of the article, the authors noted that they had made an error regarding certain data in their manuscript. The error relates to the calculations made in Table IV.

On page 88 of our article, in the left-handed column, we identified an erroneous statistical result with respect to the percentages calculated in Table IV. Please see the corrected table below.

**Table VI:** Presence or absence of residual DCIS and MRI diagnoses.

	Residual DCIS (%)		
Response	Absence	Presence	Total (%)
cCR	36 (36.7)	8 (8.2)	44 (44.9)
Residual tumor	30 (30.6)	24 (24.5)	54 (55.1)
Total	66 (67.3)	32 (32.7)	98 (100.0)

P=0.0058. cCR, clinical complete response; DCIS, ductal carcinoma *in situ*; MRI, magnetic resonance imaging.

